# British Association for the Advancement of Science

**Published:** 1837-10

**Authors:** 


BRITISH ASSOCIATION FOR THE ADVANCEMENT OF SCIENCE.

THE seventh annual meeting of this most important and most flourishing Society took place this year at Liverpool. The General Committee met on Saturday the 9th September, but the proper business of the Association did not take place till Monday the 11th: the meetings were continued daily throughout the week. On the present occasion the meeting has been most successful, upwards of 1,700 members having been enrolled; a much greater number than were present at the Bristol meeting last year. The quantity of our other matter has only left us room for the names of the officers of the Medical Section, and for the titles of the principal papers read in this. We the less regret being unable to give a full report of the latter, as we shall have an opportunity, on the publication of the next volume of the Transactions of the Association, of noticing them more in detail.

*Section* E. ANATOMY AND MEDICINE.

*President:* William Clark, M.D. *Vice-Presidents:* James Carson, M.D. F.R.S., P. M. Roget, M.D. Sec. R.S., R. Bickersteth, Esq., R. T. Evanson, M.D. M.R.I.A. *Secretaries:* J. Carson, Jun. M.D., I. R. W. Voss, M.D. *Committee:* Neil Arnott, M.D. F.R.S., Richard Bright, M.D. F.R.S., Hugh Carlyle, M.B., James Copland, M.D. F.R.S., R. T. Evanson, M.D., R. Formby, M.D., A. B. Granville, M.D. F.R.S., John Houston, M.D. M.R.I.A., James Johnson, M.D., James Macartney, M.D. F.R.S., Sir J. Murray, M.D., John Mackintosh, M.D., C. H. Orpen, M.D., W. H. Porter, Esq., C. B. Williams, M.D. F.R.S., John Yelloly, M.D. F.R.S.

Papers read on Monday:—1. "Second Report of the Sub-Committee appointed to investigate the Motions and Sounds of the Heart;" by Dr. C. Williams. 2. "On the Physical and Chemical Characters of Expectoration in different Diseases of the Lungs, with some preliminary Remarks on the Albuminous Principles existing in the Blood;" by Mr. Brett.

Papers read on Tuesday:—1. "On the Cause of Death from a Blow on the Stomach, with Remarks on the best Means calculated to restore Animation suspended by such Accident;" by Dr. Calvert Holland. 2. "Observations on the Structure of the Sacrum in Man, and in some of the lower Classes of Animals;" by Hugh Carlyle, M.B. 3. "An experimental Investigation into the Glosso-pharyngeal, Pneumogastric, and Spinal Accessory Nerves;" by John Reid, M.D. 4. "An Account of the late Influenza at Bolton, in January, February, and March, 1837; accompanied by Statistical Observations;" by James Black, M.D.

Papers read on Wednesday:—1. "Experiments on the Connexion between Nerves and Muscles;" by Dr. W. Harris Madden. 2. "On the Order of Succession of the Motions of the Heart;" by O'Bryen Bellingham, M.D. 3. "Observations on the Disease called Cocabâe by the Africans, or the Arabian Leprosy; the Ara-apatta of the Caribbees of Guiana; the Radesyge of Northern Europe; all of which appear to be identical, &c. &c.;" by John Hancock, M.D.

Papers read on Thursday:—1. "Causes and Treatment of Curvatures of the Spine, with a Description of an Apparatus for the use of Persons affected with the Disease;" by S. Hare, Esq. 2. "An Inquiry into the Influence of the Mind on the Heart and other Organs in Health and Disease;" by Dr. C. Holland.

Papers read on Friday:—1. "Some Remarks on the Crania of the Mound Indians of the Interior of North America, as compared with the Crania of the South American Indians of Peru;" by Dr. Warren. 2. "On the Morbid Anatomical Appearances of some Cases of Cholera;" by Dr. Mackintosh. 3. "On some Points in the Physiology of the Brain;" by Dr. Carson.

The next meeting of the Association takes place at Newcastle upon Tyne. The following are the officers appointed:—The Duke of Northumberland, president; the Earl of Durham, the Rev. Vernon Harcourt, and P. J. Selby, Esq. vice-presidents; Professor Peacock, and R. Murchison, Esq., general secretaries; Professor Phillips, assistant general secretary; John Adamson and W. Hutton, Esqs., of Newcastle, and Professor Johnstone, of Dublin University, local secretaries; J. Taylor, Esq., of London, general treasurer; the Rev. W. Turner, and Charles John Bigge, Esq., Newcastle, local treasurers.

---

